# Snapshot Multi-Wavelength Birefringence Imaging

**DOI:** 10.3390/s24165174

**Published:** 2024-08-10

**Authors:** Shuang Wang, Xie Han, Kewu Li

**Affiliations:** 1 School of Data Science and Technology, North University of China, Taiyuan 030051, China; 20200150@nuc.edu.cn (S.W.); hanxie@nuc.edu.cn (X.H.); 2 Engineering and Technology Research Center of Shanxi Province for Opto-Electric Information and Instrument, North University of China, Taiyuan 030051, China; 3 School of Electrical and Control Engineering, North University of China, Taiyuan 030051, China

**Keywords:** birefringence measurement, snapshot imaging, multi-wavelength channels, retardance and fast-axis azimuth

## Abstract

A snapshot multi-wavelength birefringence imaging measurement method was proposed in this study. The RGB-LEDs at wavelengths 463 nm, 533 nm, and 629 nm were illuminated with circularly polarized light after passing through a circular polarizer. The transmitted light through the birefringent sample was captured by a color polarization camera. A single imaging process captured light intensity in four polarization directions (0°, 45°, 90°, and 135°) for each of the three RGB spectral wavelength channels, and subsequently measured the first three elements of Stokes vectors (*S*_0_, *S*_1_, and *S*_2_) after the sample. The birefringence retardance and fast-axis azimuthal angle were determined simultaneously. An experimental setup was constructed, and polarization response matrices were calibrated for each spectral wavelength channel to ensure the accurate detection of Stokes vectors. A polymer true zero-order quarter-wave plate was employed to validate measurement accuracy and repeatability. Additionally, stress-induced birefringence in a PMMA arch-shaped workpiece was measured both before and after the application of force. Experimental results revealed that the repeatability of birefringence retardance and fast-axis azimuthal angle was better than 0.67 nm and 0.08°, respectively. This approach enables multispectral wavelength, high-speed, high-precision, and high-repeatability birefringence imaging measurements through a single imaging session.

## 1. Introduction

Birefringence commonly manifests in anisotropic materials, encompassing crystals, glass, plastics, thin films, and biological samples [1,2]. Additionally, external influences such as force, heat, electric, and magnetic fields can induce anisotropic properties [3,4,5,6]. The primary optical effect of birefringence entails the splitting of incident light into two orthogonal vibration directions along the principal axes. This separation leads to disparate propagation velocities for the ordinary and extraordinary light components, consequently causing a retardation effect. The retardance value and the direction of the fast axis comprehensively characterize birefringence. Birefringence measurements not only elucidate the anisotropic parameters of optical materials but also serve as the primary basis for evaluating external physical parameters. Crystal birefringence across a broad spectral dynamic range was employed for the precise calibration of polarization optics, such as waveplates and compensators [7]. Moreover, birefringence served as the foundation for essential parameters including stress defects, mechanical strength, thermal stability, imaging quality, and beam transmission quality [8]. In the realm of biological samples, birefringence played a pivotal role in biosensing and pathology assessment [9,10]. Furthermore, the multi-wavelength birefringence exhibited by novel nanostructured materials such as meta-materials and meta-surfaces offers crucial insights for the development of innovative multifunctional devices [11,12,13,14]. These applications increasingly necessitate highly accurate, real-time, and distributed measurements of birefringence across a broad spectral dynamic range.

Numerous methods for measuring birefringence have been employed, with photoelasticity techniques utilizing polariscopes, Senarmont compensation, or Tardy phase shifting being particularly prevalent [15,16,17]. These methods offer the advantage of a straightforward instrument structure and a sizable measurement aperture and have reached a state of mature commercialization. However, many photoelasticity techniques necessitate the mechanical rotation of either the polarizing element or the sample for concurrent measurement of birefringence retardance and direction. Such mechanical rotation not only diminishes measurement accuracy due to beam mechanical drift but also reduces measurement speed. More recently, liquid crystal variable retarders (LCVRs) have emerged as alternatives for birefringence measurements, heralding the development of novel photoelasticity techniques and demonstrating promise for multi-wavelength and wide dynamic range birefringence assessments [18,19]. LCVRs themselves are birefringence dispersive. Their calibration for multi-wavelength applications proves intricate. Moreover, the liquid crystal molecule orientation within LCVRs is markedly influenced by ambient temperature variations, posing challenges to the long-term stability and reproducibility of this measurement approach. Overcoming these hurdles presents formidable tasks.

Modulators such as Faraday rotator, electro-optical modulator, and photoelastic modulator were utilized to facilitate the detection of optical signal modulation, thereby achieving birefringence measurements with a precision surpassing 1 nm and a time resolution in the millisecond range [20,21]. However, polarization modulation methods are predominantly employed for single-point measurements. To obtain birefringence mappings, scanning of samples is necessary, with the dynamic range typically limited to half of the wavelength of the detection light source. Advancements in micro/nano processing technology have led to the utilization of micrometer-level wire-grid polarizers for processing imaging detector pixels. Sony Corporation, for instance, has developed an array polarization CMOS image sensor. This high-speed polarization image sensor has been effectively employed for birefringence distribution measurements, as well as rapid birefringence process assessments for fluid samples [22,23]. Presently, the polarization imaging sensor is available in monochromatic as well as color configurations. However, this image sensor is only capable of linearly polarized light detection in four polarization directions (0°, 45°, 90°, and 135°), and it does not detect circular polarization, thus constraining the birefringence retardance measurement range within the half-wavelength detection limit. Consequently, birefringence distinguishing between fast and slow axes are not achievable.

To address these limitations, this study employed an integrated approach utilizing multi-wavelength LED light arrays, in conjunction with circular polarizers to constitute a detection light source. Additionally, a color polarization camera was utilized for imaging measurements. This integrated setup was anticipated to enable snapshot multi-wavelength birefringence imaging measurements while simultaneously achieving high precision and fast measurements of birefringence retardance and fast-axis azimuthal angle across a wide dynamic range. Such advancements provide sophisticated means for the measurement of birefringence dispersion and properties of novel materials.

## 2. Methods

**Figure 1:**
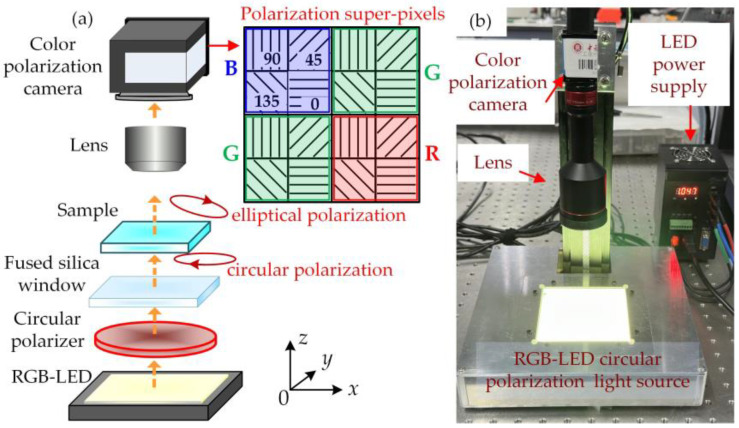
Birefringence imaging system. Schematic (**a**) and device (**b**) diagram.

To address the issue of existing polarization image sensors’ insensitivity to circular polarization, this study modified the detection light source to emit circularly polarized light. As depicted in Figure 1a, an integrated red, green, and blue multi-wavelength LED light source (RGB-LED) was transformed into circularly polarized light through a circular polarizer. This circularly polarized light was then directed onto the sample for snapshot imaging measurements using a polarization camera.

**Figure 2:**
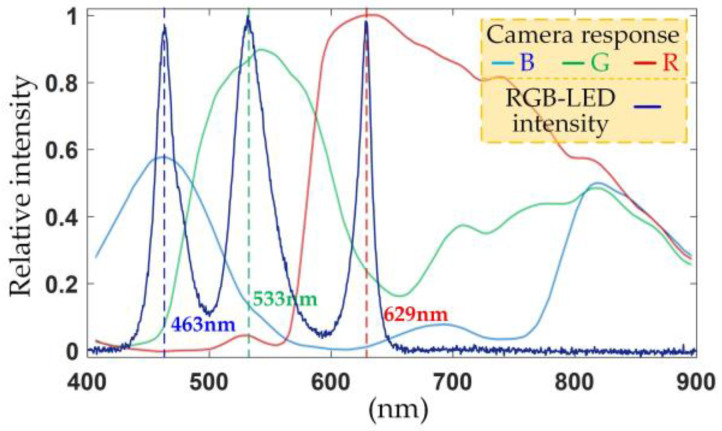
RGB-LED spectrum.

As illustrated in Figure 1b, the camera utilized in this study was a color polarization camera model DYK 33GX250 from The Imaging Source Asia Co., Ltd., (Shenzhen, China), featuring a Sony IMX250MYR-C CMOS (color) image sensor. The camera boasted a resolution of 2448 × 2048 pixels (5 MP) with a pixel size of 3.45 µm × 3.45 µm and operated at a frame rate of 75 fps. The camera employed a telecentric lens (model FT03X110, Hikvision Digital Technology Co., Ltd., Hangzhou, China), providing a field of view for the object of 36.67 mm and a working distance of 110 mm. The camera’s imaging system comprised a polarized superimage element formed by four wire-grid polarizer pixels oriented horizontally (0°), vertically (90°), and diagonally (45° and 135°). Each polarized superimage element was equipped with a mosaic of Bayer filters in a sequential arrangement, as depicted in the inset of Figure 1a. The RGB-LEDs utilized in the setup were sourced from KOMA Vision Technology Co., Ltd., (Dongguan, China), illuminating a surface area of 100 × 100 mm. The LEDs emit natural light. A circular polarizer was fabricated by laminating a linear film polarizer onto a high-contrast polymer achromatic quarter-wave retarder sheet, covering the wavelength range of 400–700 nm, sourced from Edmund Optics (Shenzhen, China) Co., Ltd. The ellipticity uniformity of the circularly polarized light passing through the polarizer exceeds 99%. To shield the light source between the LEDs and the sample, a fused silica window measuring 100 × 100 mm with a thickness of 18 mm was installed. This window protects the circular polarizer from scratches and contamination during sample measurement while providing a smooth, light-transmitting support platform for the sample. The spectral response characteristics of the color camera were taken into account when configuring the RGB three-color light source in this setup. Concurrent illumination with the RGB light sources was confirmed through spectrogram measurements conducted using a spectrometer (model HR4000CG-UV-NIR, Ocean Optics, Orlando, FL, USA), as presented in Figure 2.

The RGB-LED employed in this study emitted light at three distinct wavelengths: 463 nm, 533 nm, and 629 nm. Following the passage through the circular polarizer, the light emitted by the RGB-LED was transformed into a circularly polarized illumination source. Due to the ellipticity uniformity exceeding 99%, the difference between the intensity of the circularly polarized light component and the total light intensity is less than 1%. This transformation can be characterized using the Stokes vector as $S_{\mathrm{in}(\lambda )}=I_{\mathrm{in}(\lambda )}{\left[\begin{array}{cccc} 1 & 0 & 0 & 1 \end{array}\right]}^{T}$, where $I_{\mathrm{in}(\lambda )}$ represents the intensity of circularly polarized light at the wavelength λ. The polarization transmission characteristics of the sample can be described using the Mueller matrix [24] as follows:

(1) $$M_{S}=\left[\begin{array}{cccc} \begin{array}{c} 1\\ 0\\ 0\\ 0 \end{array} & \begin{array}{c} 0\\ \cos^{2}(2\rho )+\sin^{2}(2\rho )\cdot \cos(X)\\ \sin(4\rho )\cdot \sin^{2}(X/2)\\ \sin(2\rho )\sin(X) \end{array} & \begin{array}{c} 0\\ \sin(4\rho )\cdot \sin^{2}(X/2)\\ \sin^{2}(2\rho )+\cos^{2}(2\rho )\cdot \cos(X)\\ -\cos(2\rho )\sin(X) \end{array} & \begin{array}{c} 0\\ -\sin(2\rho )\sin(X)\\ \cos(2\rho )\sin(X)\\ \cos(X) \end{array} \end{array}\right]$$

In the aforementioned equation, ρ denotes the fast-axis azimuthal angle of birefringence, and *X* represents the phase retardance corresponding to the birefringence within the sample. This phase retardance can be expressed for various detection light wavelength, as $X_{\left(\lambda \right)}=2\pi R/\lambda =2\pi \Delta n_{\left(\lambda \right)}d/\lambda$. *R* represents the retardance. The dispersion in the birefringence of the sample for different detection light wavelengths is represented by $\Delta n_{\left(\lambda \right)}$, and *d* indicates the thickness of the sample. Upon transmission through the sample, the circularly polarized light is transformed into elliptically polarized light. This elliptically polarized light is described by the Stokes vector as $S_{\mathrm{out}(\lambda )(i)}=M_{S}S_{\mathrm{in}(\lambda )}$. The circularly polarized light $S_{\mathrm{in}(\lambda )}$ after passing through the sample is defined in Equation (1). The resulting elliptically polarized light transmitted through the sample is then solved as follows:

(2) $$S_{\mathrm{out}(\lambda )(i)}=I_{\mathrm{in}(\lambda )}\left[\begin{array}{c} 1\\ -\sin(2\rho )\sin(X)\\ \cos(2\rho )\sin(X)\\ \cos(X) \end{array}\right]$$

Utilizing a polarization camera for single imaging detection, each polarized superimage element captured the light intensity parameters in four polarization directions: horizontal (0°), vertical (90°), and two diagonal (45° and 135°), denoted as $I_{0}$, $I_{45}$, $I_{90}$, and $I_{135}$, respectively. The first three components of the Stokes vector, representing orthogonal radiation intensity, the difference between horizontal and vertical intensities, and the disparity between the two diagonal intensities, were concurrently measured. Therefore, the polarization camera detected the elliptically polarized light transmitted through the sample, expressed as $S_{\mathrm{out}(\lambda )}=M{\left[\begin{array}{cccc} I_{0} & I_{45} & I_{90} & I_{135} \end{array}\right]}^{T}$, where $M_{\mathrm{ij}}$ = [0.5, 0.5, 0.5, 0.5; 1, 0, −1, 0; 0, 1, 0, −1] is the polarization response matrix. The light intensity acquired from each polarized superimage element satisfies the following relation:

(3) $$\left\{\begin{array}{c} S_{0(\lambda )}=\frac{I_{0(\lambda )}+I_{90(\lambda )}+I_{45(\lambda )}+I_{135(\lambda )}}{2}=I_{\mathrm{in}(\lambda )}\\ S_{1(\lambda )}=I_{0(\lambda )}-I_{90(\lambda )}=-I_{\mathrm{in}(\lambda )}\sin(2\rho )\sin(X)\\ S_{2(\lambda )}=I_{45(\lambda )}-I_{135(\lambda )}=I_{\mathrm{in}(\lambda )}\cos(2\rho )\sin(X) \end{array}\right.$$

From Equation (3), two relative light intensity values were further obtained as in Equation (4).

(4) $$\left\{\begin{array}{c} \gamma_{I(\lambda )}={\left(\frac{S_{1(\lambda )}}{S_{0(\lambda )}}\right)}^{2}+{\left(\frac{S_{2(\lambda )}}{S_{0(\lambda )}}\right)}^{2}=\sin^{2}(X_{\left(\lambda \right)})\\ \gamma_{\mathrm{II}(\lambda )}=-\frac{S_{1(\lambda )}}{S_{2(\lambda )}}=\tan(2\rho_{\left(\lambda \right)}) \end{array}\right.$$

**Figure 3:**
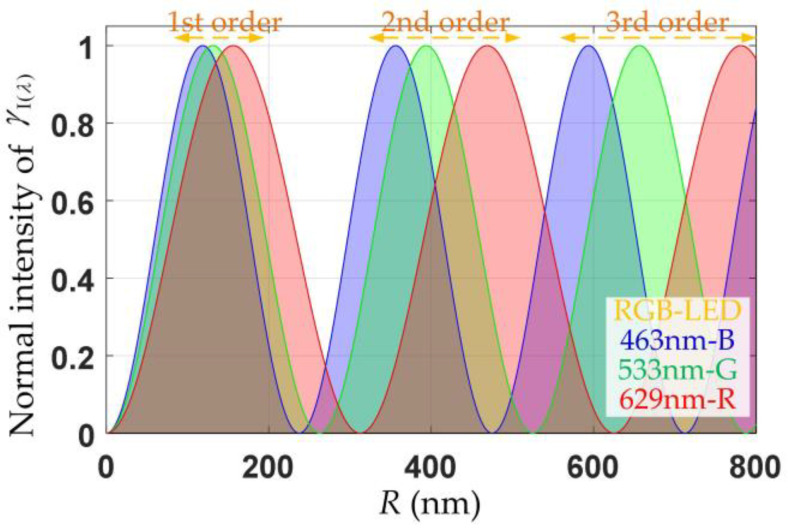
Normalized intensity $\gamma_{I(\lambda )}$ vs. retardance *R*.

The numerical simulation depicting the variation in normal intensity in relation to the retardance value $R=\lambda X_{\left(\lambda \right)}/2\pi$ across the three RGB wavelength channels, 463 nm, 533 nm, and 629 nm, was shown in Figure 3.

In Figure 3, the relationship between normal intensity $\gamma_{I(\lambda )}$ and retardance *R* was periodic. The three RGB wavelengths displayed distinct patterns of transmitted light intensity at identical values of retardance, while varying levels of retardance exhibited graded characteristics. By employing Equation (4), the phase retardance *X* and the fast-axis azimuthal angle ρ of the sample were concurrently determined.

(5) $$\left\{\begin{array}{c} X_{\left(\lambda \right)}=\arcsin(\pm \sqrt{\gamma_{I(\lambda )}})\\ \rho_{\left(\lambda \right)}=\frac{1}{2}\arctan(\gamma_{\mathrm{II}(\lambda )}) \end{array}\right.$$

The phase retardation could be expressed further through the normal intensity $\gamma_{I(\lambda )}$ for the first- and second-order light intensities, as depicted in Figure 3.

(6) $$X_{\left(\lambda \right)}=\left\{\begin{array}{c} \arcsin(\sqrt{\gamma_{I(\lambda )}})\begin{array}{c} \begin{array}{cc} & R\in [0,\lambda /4) \end{array} \end{array}\\ \pi -\arcsin(\sqrt{\gamma_{I(\lambda )}})\begin{array}{cc} & R\in [\lambda /4,\lambda /2) \end{array}\\ \pi +\arcsin(\sqrt{\gamma_{I(\lambda )}})\begin{array}{cc} & R\in [\lambda /2,3\lambda /4) \end{array}\\ 2\pi -\arcsin(\sqrt{\gamma_{I(\lambda )}})\begin{array}{cc} & R\in [3\lambda /4,\lambda ] \end{array} \end{array}\right.$$

For the majority of optical materials, birefringent dispersion is typically minimal [25], and the variance in birefringence retardance across shorter spectral ranges is sufficiently minute to serve as a constraint for precise retardance determination at each wavelength. Moreover, the range of the fast-axis azimuthal angle can be ascertained concurrently with retardance determination. When R∈(0,λ/2), according to light intensity described in Equation (3), whether the light intensity is positive or negative, the range of fast-axis azimuthal angle can be determined as follows:

(7) $$\rho_{\left(\lambda \right)}=\left\{\begin{array}{c} \frac{1}{2}\arctan(\gamma_{\mathrm{II}(\lambda )})\begin{array}{c} \begin{array}{cc} \in [0,45^{\circ }) & S_{1(\lambda )}\leq 0,S_{2(\lambda )}>0 \end{array} \end{array}\\ \frac{\pi }{2}+\frac{1}{2}\arctan(\gamma_{\mathrm{II}(\lambda )})\begin{array}{cc} \in [45^{\circ },90^{\circ }] & S_{1(\lambda )}\leq 0,S_{2(\lambda )}\leq 0 \end{array}\\ \begin{array}{cc} \frac{\pi }{2}+\frac{1}{2}\arctan(\gamma_{\mathrm{II}(\lambda )})\in (90^{\circ },135^{\circ }] & S_{1(\lambda )}>0,S_{2(\lambda )}\leq 0 \end{array}\\ \begin{array}{cc} \pi +\frac{1}{2}\arctan(\gamma_{\mathrm{II}(\lambda )})\in (135^{\circ },180^{\circ }] & S_{1(\lambda )}\geq 0,S_{2(\lambda )}>0 \end{array} \end{array}\right.$$

When R∈(λ/2,λ), according to light intensity described in Equation (3), the range of fast-axis azimuthal angle can be determined as follows:

(8) $$\rho_{\left(\lambda \right)}=\left\{\begin{array}{c} \frac{1}{2}\arctan(\gamma_{\mathrm{II}(\lambda )})\begin{array}{c} \begin{array}{cc} \in [0,45^{\circ }) & S_{1(\lambda )}\geq 0,S_{2(\lambda )}<0 \end{array} \end{array}\\ \frac{\pi }{2}+\frac{1}{2}\arctan(\gamma_{\mathrm{II}(\lambda )})\begin{array}{cc} \in [45^{\circ },90^{\circ }] & S_{1(\lambda )}\geq 0,S_{2(\lambda )}\geq 0 \end{array}\\ \begin{array}{cc} \frac{\pi }{2}+\frac{1}{2}\arctan(\gamma_{\mathrm{II}(\lambda )})\in (90^{\circ },135^{\circ }] & S_{1(\lambda )}<0,S_{2(\lambda )}\geq 0 \end{array}\\ \begin{array}{cc} \pi +\frac{1}{2}\arctan(\gamma_{\mathrm{II}(\lambda )})\in (135^{\circ },180^{\circ }] & S_{1(\lambda )}\leq 0,S_{2(\lambda )}<0 \end{array} \end{array}\right.$$

Thus, a single imaging measurement enables the simultaneous determination of birefringence retardance and fast-axis azimuthal angle at multi-wavelengths.

## 3. Experiments

### 3.1. Camera Polarization Response Calibration

**Figure 4:**
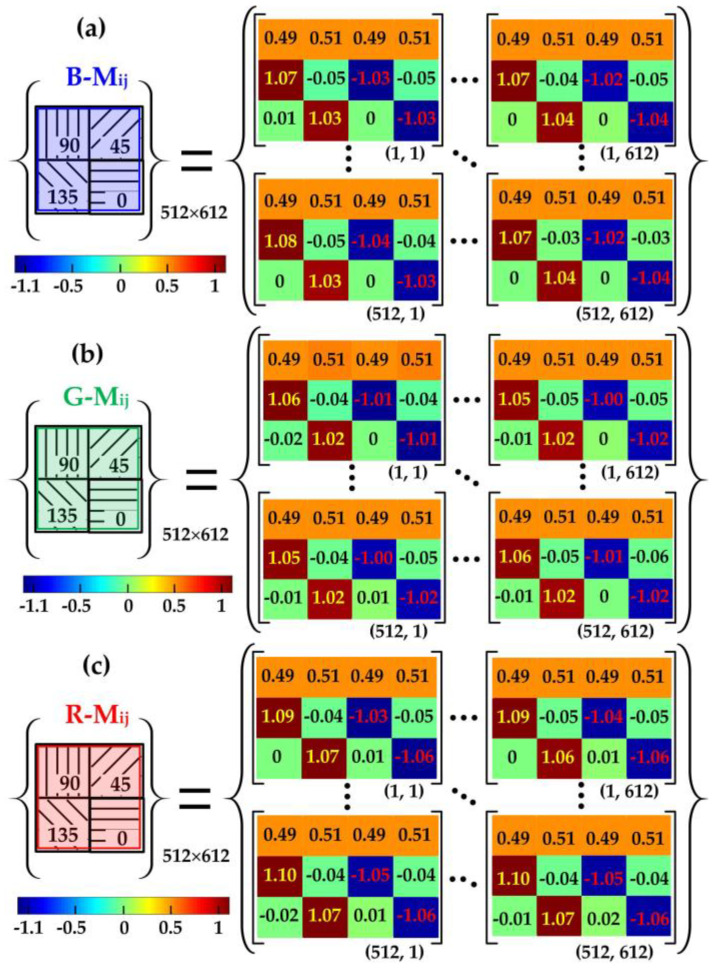
Calibration of response matrices: (**a**) B-, (**b**) G-, and (**c**) R-color response matrices.

The precise calibration of the camera’s polarization response is crucial due to several factors: the non-uniform photoelectric response exhibited by the CMOS image sensor pixels and the variations in extinction ratio and transmittance among the wire-grid polarizer pixels. Initially, the circular polarizer was removed from the detection light source. Subsequently, without introducing any sample, a linear polarizer was positioned between the RGB-LED and the polarization camera. The calibration procedure followed the methodology delineated in reference [26]. The angle of the transmittance axis (*θ*) was systematically adjusted from 0° to 180° in 2° increments. Calibration was carried out to derive the response matrices for each polarized superimage element, as illustrated in Figure 4a–c.

**Figure 5:**
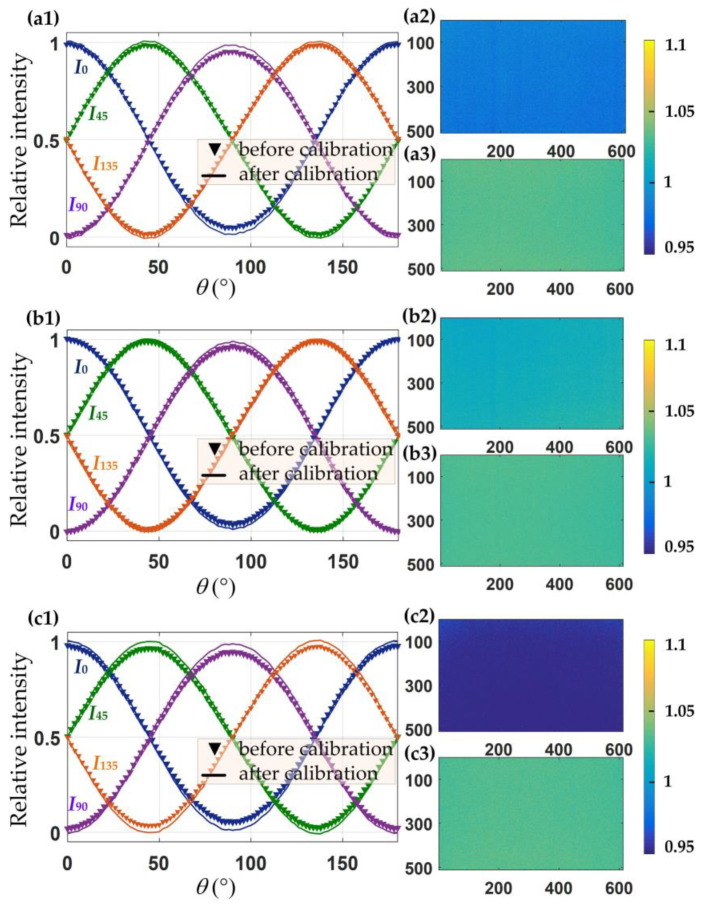
Measured light intensity before and after calibration: (**a1**) B-, (**b1**) G-, and (**c1**) R-color channels. Degree of linear polarization: (**a2**) B-, (**b2**) G-, and (**c2**) R-color channels before calibration and (**a3**) B-, (**b3**) G-, and (**c3**) R-color channels after calibration for 0° direction of polarizer.

Based on the findings presented in Figure 4, the response matrices for each polarized superimage element were calibrated for the RGB color polarization camera. Each color polarization yielded 512 × 612 polarization superimages, with each superimage corresponding to four measurement matrix subunits containing 3 × 4 polarization directions. The calibration process effectively mitigated measurement errors in light intensity. To illustrate, the polarized light detection results before and after calibration for the three color channels are documented in Figure 5, with the pixel (256, 306) superimage element serving as an exemplar.

The results presented in Figure 5(a1–c1) indicate that the light intensity measurements were notably more accurate when employing the calibrated response matrices. For instance, considering the incident light at the 0° direction of the polarizer, the degree of linear polarization (DOLP) for the three color measurements—(a2,a3) for B-, (b2,b3) for G-, and (c2,c3) for R-color channel—before and after the calibration of the response matrices were examined. Specifically, the B-color DOLP improved from 0.972 to 1.005, the G-color DOLP improved from 0.992 to 1.003, and the R-color DOLP improved from 0.957 to 1.004. Moreover, the response fluctuation of the entire superimage arrays enhanced from 0.001 to 0.0004, thus ensuring the high accuracy of birefringence measurement applications.

### 3.2. Birefringence Measurements Conducted on a Quarter-Wave Plate and a PMMA Arch-Shaped Workpiece

The accuracy and repeatability of the system were evaluated by employing a polymer true zero-order quarter-wave plate as a standard sample. The wave plate was a QWP25-633A-M quarter-wave plate manufactured by Shenzhen LUBON Technology Co., Ltd., Shenzhen, China and designed for operation at a working wavelength of 633 nm, featuring an optical aperture diameter of 21.5 mm. According to the manufacturer’s measurement and calibration data, the retardance of the wave plate is 188.9 nm at 463 nm (B), 170.0 nm at 533 nm (G), and 157.3 nm at 629 nm (R) across the three wavelength channels [27].

**Figure 6:**
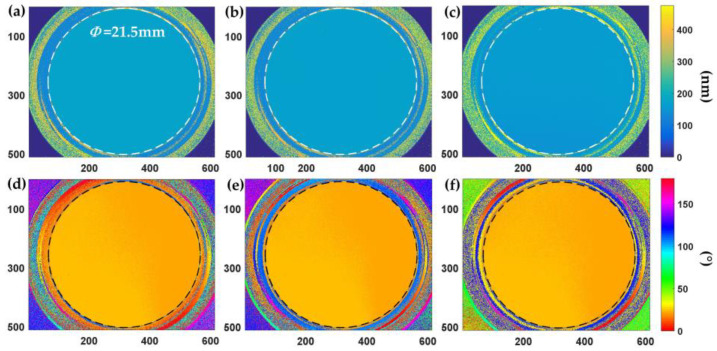
Results of quarter-wave plate: (**a**–**c**) retardance at B-, G-, and R-wavelength channels and (**d**–**f**) fast-axis azimuthal angle at B-, G-, and R-wavelength channels.

The wave plate was placed in our system, its fast-axis orientation was adjusted to approximately 22.5°, and then the measurement was started. The measurement results for the three RGB wavelength channels obtained using our system are shown in Figure 6.

In Figure 6, the retardance and fast-axis azimuthal angle measurements are uniformly distributed across the entire aperture of the wave plate. A single imaging measurement sufficed to obtain the birefringence retardance and fast-axis azimuthal angles for all three spectral wavelength channels of RGB. The average and standard deviation of the retardance and fast-axis azimuthal angle within the effective aperture area of the wave plate (horizontal: 86 to 426 pixels; vertical: 145 to 485 pixels) are presented in Table 1 for the three spectral wavelength channels. We also included the calibration data provided by the manufacturer in Table 1.

Based on the test results of the true zero-order quarter-wave plate, noticeable differences in birefringence were observed across the three wavelength channels, primarily attributable to spectral dispersion birefringence. The measurement value for the R channel was very close to the retardance value at 633 nm wavelength because their wavelengths were similar. The retardance value decreased as the wavelength increased. The maximum relative deviation between the measured values in the three spectral channels and the calibration values provided by the manufacturer is 1.1% in the R channel, calculated as |159.06 − 157.3|/157.3 = 1.1%. This indicated that the system achieved high measurement accuracy. The standard deviation of birefringence measurements did not exceed 0.67 nm in any of the spectral wavelength channels, indicating a high level of repeatability. Additionally, the fast-axis azimuthal angle remained consistent across multispectral measurements, with a standard deviation of the angle not exceeding 0.08°.

**Figure 7:**
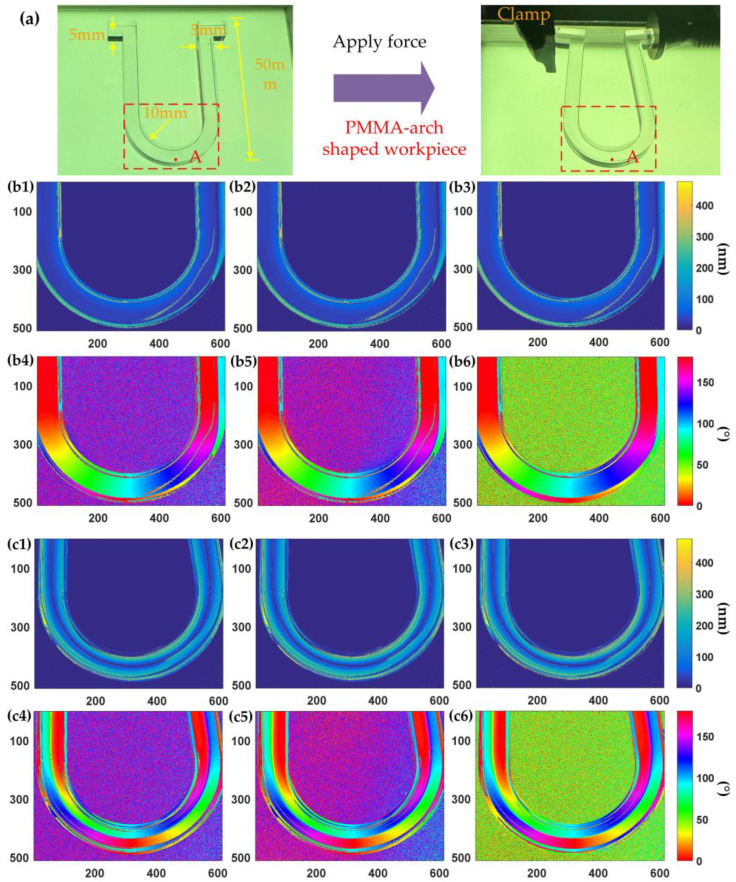
Stress birefringence measurements of PMMA arch-shaped workpiece: (**a**) workpiece, (**b1**–**b3**, **c1**–**c3**) retardance, and (**b4**–**b6**, **c4**–**c6**) fast-axis azimuthal angles at B-, G-, and R-wavelength channels before and after applying force. Point A in Figure 7(a) is a region of interest measuring about 3 × 3 pixels.

To further validate the feasibility of snapshot multispectral birefringence measurements, stress birefringence in an arch-shaped workpiece fabricated from polymethyl methacrylate (PMMA) was assessed both before and after the application of force, as depicted in Figure 7.

The PMMA arch-shaped workpiece, as depicted in Figure 7a, had a thickness of 5 mm. Birefringence distribution was measured for both unclamped and clamped states, with clamping applied at the bottom of the arch-shaped workpiece. The distribution of birefringence within the workpiece was non-uniform prior to the application of force. Figure 7(b1–b3) distinctly illustrate significant and highly heterogeneous edge stresses, primarily attributed to residual stresses resulting from the laser ablation processing of the workpiece. The birefringence retardance within the workpiece did not exceed 120 nm. Figure 7(b4–b6) show the birefringence distribution within the workpiece being divided into two sections from the midpoint of the arch. At the midpoint, the fast-axis azimuthal angle of the birefringence was approximately 90°. Subsequently, the left half of the workpiece exhibited a gradual transition from 90° to 0 in accordance with the shape of the arch, while the right half transitioned from 90° to 180° following the arch’s curvature.

Figure 7(c1–c3) clearly illustrate an increase in birefringence following the application of force. However, there was a smaller region of birefringence distribution along the midpoint of the arch. Consequently, the arch exhibited a layered sub-regional arrangement along its inner and outer edges. Notably, the fast-axis azimuthal angle of birefringence at the midpoint of the outer gable arch approached 0, and the intersection point at 90° shifted to where the arch intersects the vertical straight line. Meanwhile, the inner 90° remained centrally positioned. We recorded the change in birefringence value at the outer eave vertex of the arched workpiece, approximately 1 mm from the region of interest designated as point A. The region of interest measuring about 3 × 3 pixels was recorded for further analysis. Birefringence measurements are detailed in Table 2.

From the statistical data recorded in Table 2, it was evident that the birefringence value at the region of interest, point A, on the arch-shaped workpiece increased following the application of external force. Additionally, there was a discernible dispersion in the distribution of birefringence retardance across the three spectral wavelength channels. Notably, the direction of the fast-axis distribution shifted from its original vertical orientation by approximately 90° to a horizontal orientation of about 0° (180°).

## 4. Discussion

Through the precise calibration of the response matrix for each polarized superimage element, the polarization directions (0°, 45°, 90°, and 135°) of light intensity were accurately detected across the three RGB wavelength channels (463 nm, 533 nm, and 629 nm) in a single imaging session. This calibration facilitated highly accurate and sensitive birefringent snapshot imaging measurements. The measurement process, exemplified by the true zero-order polymer quarter-wave calibration, resulted in standard deviation values for birefringence retardance and fast-axis azimuthal angle of better than 0.67 nm and 0.08°, respectively, indicating high repeatability and sensitivity. The birefringence distribution of materials such as wave-plates and PMMA could be rapidly assessed using the snapshot imaging techniques. The RGB three-channel birefringence retardance measurements exhibited spectral dispersion characteristics. However, the directional distribution of birefringence fast-axis azimuthal angle across the three-channel measurements remained consistent and did not follow spectral dispersion patterns. Given the camera’s frame rate of 75 fps, the measurement time could be as short as 13 ms if adequate light intensity for illumination is provided. In addition, the lens utilized in this investigation had a field of view of 36.67 mm. Enhancing the system’s lens with a broader field of view would enable an extension of the aperture for birefringence imaging detection. The test results of this study are compared with those in the existing literature and are listed in Table 3.

This approach enabled high-precision and high-repeatability birefringence imaging measurements across three wavelength channels in a single imaging session. In the single-wavelength channel measurement method, the birefringence retardance range is limited to 0–λ/2, whereas multi-wavelength measurements can extend the retardance measurement range to 0–λ. This multi-wavelength measurement technique is applicable to the study of material birefringence dispersion. The snapshot single-imaging birefringence measurement achieved in this work makes the method particularly suitable for measuring high-speed dynamic changes in birefringence distribution.

## 5. Conclusions

In summary, a study focusing on birefringence imaging measurements in RGB multi-wavelength channels was conducted. The experimental setup included an RGB-LED array incorporating a circular polarizer and a fused silica window, which generated a uniform circularly polarized surface-array detection light source for illuminating the sample. The outgoing light was then captured by a color polarized camera. Imaging measurements of the outgoing light (*S*_0_, *S*_1_, and *S*_2_) were performed through a single measurement of light intensity across the polarization directions of the three RGB spectral channels (0°, 45°, 90°, and 135°). The birefringence retardance and fast-axis azimuthal angle were determined through a combination of measurements from the three wavelength channels simultaneously. We conducted a thorough analysis of the measurement principle and constructed the experimental system devices. In the experiments, we initially completed the precise calibration of the measurement response matrices of the polarization camera across the RGB channels. Subsequently, the scheme’s measurement accuracy and repeatability were demonstrated using the polymer true zero-order quarter-wave plate. We further investigated the stress birefringence distribution before and after applying force to the PMMA workpiece. The experimental results indicated standard deviations of birefringence retardance and fast-axis azimuthal angle better than 0.67 nm and 0.08°, respectively, affirming the high level of repeatability and sensitivity. The method showcased that a single imaging measurement could effectively capture the birefringence distribution across multiple-wavelength channels of RGB. The achieved multispectral, high-speed, high-precision, and high-repeatability birefringence imaging measurements offer an effective method for conducting birefringence assessments of optical materials and components. Additionally, they facilitate analysis and evaluation of dispersion.

## Figures and Tables

**Table 1:** Quarter-wave plate experimental data.

Wavelength Channel	Birefringence Retardance			Fast-Axis Azimuthal Angle	
	Mean Value (nm)	Standard Deviation Value (nm)	Manufacturer’s Calibration Values (nm)	Mean Value (°)	Standard Deviation Value (°)
463 nm-B	188.57	0.67	188.9	22.55	0.06
533 nm-G	168.72	0.58	170.0	22.53	0.06
629 nm-R	159.06	0.45	157.3	22.54	0.08

**Table 2:** Birefringence measurements.

Wavelength Channel	Before Applying Force		After Applying Force	
	Retardance (nm)	Fast-Axis Azimuthal Angle (°)	Retardance (nm)	Fast-Axis Azimuthal Angle (°)
463 nm-B	34.86	93.02	247.51	177.87
533 nm-G	32.19	93.02	242.06	177.91
629 nm-R	31.90	92.87	237.55	177.42

**Table 3:** Comparison with reported birefringence measurement methods.

Method	Standard Deviation of Retardance	Standard Deviation of Fast-Axis Azimuthal Angle	Wavelength Channel	Birefringence Distribution Measurement	Ref.
Senarmont compensation	±5 nm	0.1°	1	multiple scanning	[16]
LCVR	±9.6 nm	-	3	multiple imaging	[19]
photoelastic modulation	0.03 nm	0.02°	1	multiple scanning	[21]
polarization CMOS	0.005 rad (0.42 nm @ 526 nm)	0.1°	1	single imaging	[22]
RGB-LED + circular polarizer + color polarized camera	0.67 nm	0.08°	3	single imaging	This work

## Data Availability

Data are contained within the article.
